# The different facets of organelle interplay—an overview of organelle interactions

**DOI:** 10.3389/fcell.2015.00056

**Published:** 2015-09-25

**Authors:** Michael Schrader, Luis F. Godinho, Joseph L. Costello, Markus Islinger

**Affiliations:** ^1^Department of Biosciences, College of Life and Environmental Sciences, University of ExeterExeter, UK; ^2^Centre for Cell Biology and Department of Biology, University of AveiroAveiro, Portugal; ^3^Neuroanatomy, Center for Biomedicine and Medical Technology Mannheim, University of HeidelbergMannheim, Germany

**Keywords:** membrane contact sites, organelle dynamics, peroxisomes, mitochondria, endoplasmic reticulum, intracellular signaling, MAM, PAM

## Abstract

Membrane-bound organelles such as mitochondria, peroxisomes, or the endoplasmic reticulum (ER) create distinct environments to promote specific cellular tasks such as ATP production, lipid breakdown, or protein export. During recent years, it has become evident that organelles are integrated into cellular networks regulating metabolism, intracellular signaling, cellular maintenance, cell fate decision, and pathogen defence. In order to facilitate such signaling events, specialized membrane regions between apposing organelles bear distinct sets of proteins to enable tethering and exchange of metabolites and signaling molecules. Such membrane associations between the mitochondria and a specialized site of the ER, the mitochondria associated-membrane (MAM), as well as between the ER and the plasma membrane (PAM) have been partially characterized at the molecular level. However, historical and recent observations imply that other organelles like peroxisomes, lysosomes, and lipid droplets might also be involved in the formation of such apposing membrane contact sites. Alternatively, reports on so-called mitochondria derived-vesicles (MDV) suggest alternative mechanisms of organelle interaction. Moreover, maintenance of cellular homeostasis requires the precise removal of aged organelles by autophagy—a process which involves the detection of ubiquitinated organelle proteins by the autophagosome membrane, representing another site of membrane associated-signaling. This review will summarize the available data on the existence and composition of organelle contact sites and the molecular specializations each site uses in order to provide a timely overview on the potential functions of organelle interaction.

## Introduction

**Figure 1:**
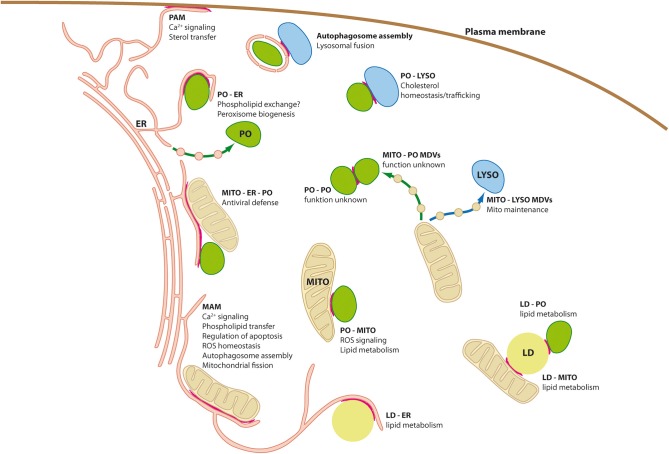
**Organelle interplay and interorganellar contacts**. Schematic diagram of a mammalian cell depicting organelle interplay and interorganellar membrane contacts (highlighted by red lines). ER, endoplasmic reticulum; LD, lipid droplets; LYSO, lysosome; MAM, mitochondria associated-membrane; MDVs, mitochondria derived vesicles; MITO, mitochondrium; PAM, plasma membrane-associated membrane; PO, peroxisome.

**Figure 2:**
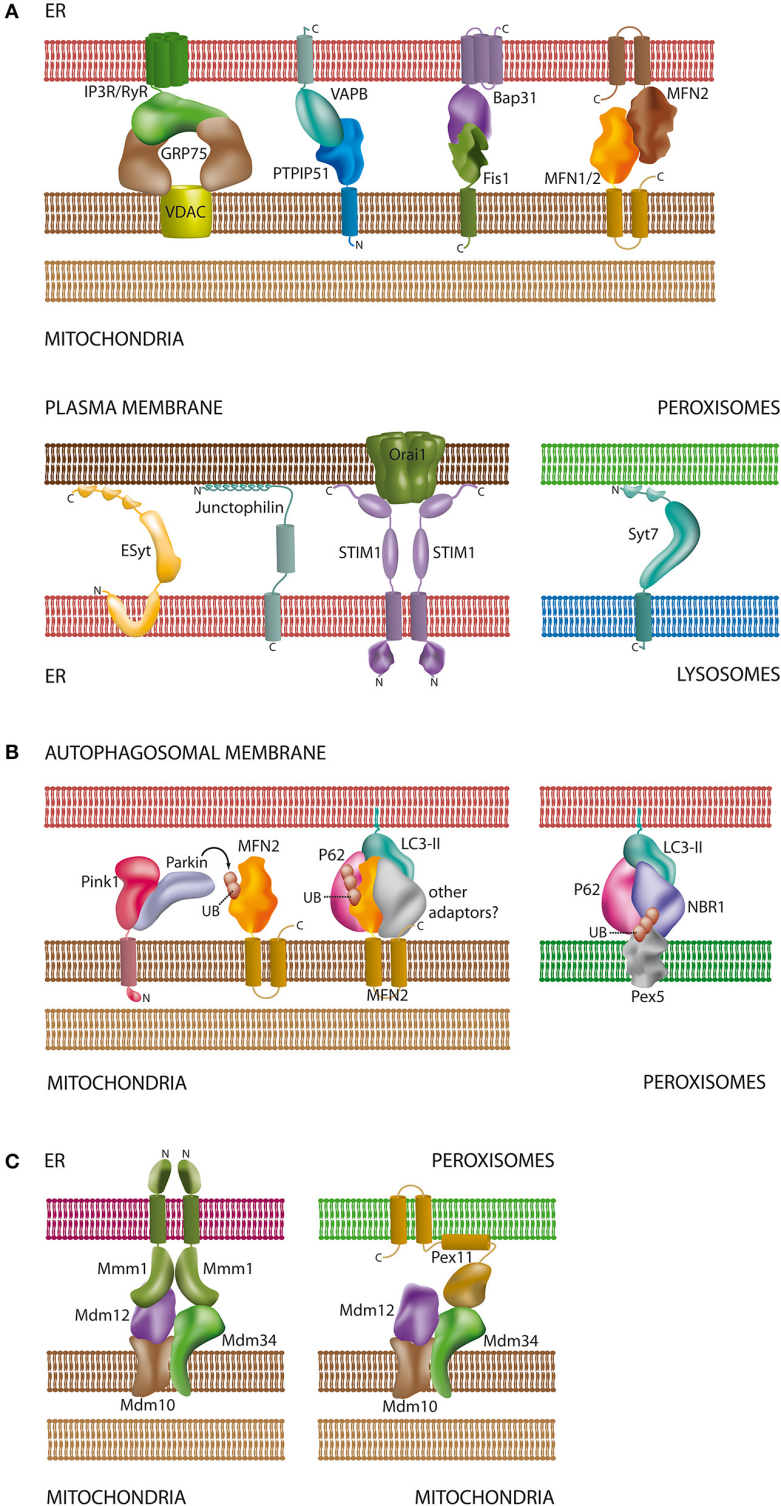
**Schematic overview of proteins and lipids involved in the interaction of organelles**. **(A)** Tethering complexes in mammals: unlike in yeast species only a few protein complexes have been characterized at the molecular level and involve protein-protein and protein-lipid contacts [see Sections Connections between the ER and the Plasma Membrane, The Mitochondria-associated Membrane of the ER (MAM), Interplay between Peroxisomes and Mitochondria, and Lysosomal Interactions and Autophagy]. Part of the tethering complexes shown may only comprise core complexes, which will interact with additional proteins for regulatory purposes; **(B)** contacts between mitochondria/peroxisomes and the autophagosomal membrane: both organelles require ubiquitination of membrane proteins for recognition by the autophagosome. In addition to MFN2 (Mitochondria) and Pex5 (Peroxisomes) other ubiquitinated organelle proteins have been described to participate in autophagosomal contacts [see Sections The Mitochondria-associated Membrane of the ER (MAM) and Lysosomal Interactions and Autophagy]; **(C)** ERMES as a multifunctional tethering complex in yeast: unlike mammals, yeast species possess the ERMES oligomeric complex at the mitochondrial membrane. ERMES forms complexes with the ER and peroxisomes [see Sections The Mitochondria-associated Membrane of the ER (MAM) and Interplay between Peroxisomes and Mitochondria]. In addition, a considerable number of other tethering complexes (not shown) have been described in yeast (Prinz, 2014). For molecular details and references of the depicted complexes please refer to the corresponding sections of this review. Membrane spanning α-helices in the proteins are depicted as cylindrical segments; C- and N-termini are marked with the corresponding letters.

In eukaryotic cells sophisticated membrane-bound organelles have evolved which enable the cell to compartmentalize specialized biochemical reactions in specific locations within the cell (Figure 1). Historically, subcellular compartments were regarded as isolated, membrane bound biochemical entities, and individual organelles such as mitochondria, lysosomes, peroxisomes, or the endoplasmic reticulum (ER) have been associated with distinct cellular tasks including ATP production, protein degradation, lipid breakdown, and protein export. In recent years, a combination of ultrastructural studies, fluorescence-based live cell imaging techniques, molecular cell biology, biochemistry, and modern proteomics approaches has substantially changed this view towards a highly dynamic, cooperative and complex network of interacting and communicating subcellular compartments (Figure 1). It is evident that intracellular compartments have to exchange material and transmit signals between each other to maintain and balance cellular activities. Cooperative functions of organelle networks include (1) metabolic interaction, (2) intracellular signaling, (3) cellular maintenance, (4) regulation of programmed cell death/cell survival, and (5) pathogen defence. Mechanistically, functional interplay can be established by vesicular transport (as initially revealed for organelles within the secretory pathway), by exchange of metabolites or signaling molecules through diffusion, or direct physical contacts which are mediated by specialized membrane contact sites (Figure 2). It is becoming evident that the cytoskeleton and molecular motors are not the sole organizers of cellular architecture, and that membrane contacts can influence the positioning and motility of organelles. Organelle interaction also depends on the total number of organelles which is regulated by organelle biogenesis/formation, membrane dynamics and autophagic processes. Remarkably, these processes also involve membrane contact sites, for example ER-mitochondria contacts which are supposed to contribute to mitochondrial division [see Sections The Mitochondria-associated Membrane of the ER (MAM) and Interplay between Peroxisomes and Mitochondria] or interactions with lysosomes during autophagy (see Section Lysosomal Interactions and Autophagy). Membrane contact sites involve tethering of two membranes in close apposition (typically within 30 nm) and the enrichment of specific proteins and/or lipids at these sites (see Table 1). In general, the tethered membranes do not fuse, but contact formation has an impact on the function or composition of one or both organelles. Although membrane contacts between organelles have been reported in early ultrastructural studies, their important functions in intracellular signaling, metabolite transport/metabolism, organelle dynamics and transport is just beginning to emerge. Furthermore, a growing number of proteins with potential tethering functions are being identified in yeast and mammals.

**Table 1:** **Summary of the protein components found at organelle contact sites in mammalian cells**.

**Organelles**	**Contact site**	**Contact site function**	**Confirmed components**	**Putative components**	**References**
ER-PM	Junctional membrane complexes (JMC)	Various, including coupling electric excitation of the PM with myofilament contraction	Junctophilin1-4 (JP1-4): Bind PIP lipids at cytoplasmic side of PM		Takeshima et al., 2015
	Store-operated calcium entry pathway (SOCE)	Replenishment of ER Ca^2+^ levels	STIM1: ER resident, binds PIP lipids and Orai1 at plasma membrane, Orai1: PM resident, forms channel facilitating Ca^2+^ uptake.		Liou et al., 2005; Park et al., 2009
	ORPS	Generating focal lipid exchange sites	ORP1/2: Sterol transport from PM to ER		Ngo et al., 2010; Jansen et al., 2011
	Extended Synaptotagmins	Implicated in the mediation of lipid transfer	E-Syt1-3: ER proteins mediating Ca^2+^-dependent tethering of the ER to PM		Giordano et al., 2013; Fernández-Busnadiego et al., 2015
ER-MITO	MAM	Various, including lipid metabolism, Ca^2+^ signaling, and regulation of mitochondrial maintenance	PEMT: converts PE to PC	DGAT2: triglyceride synthesis, TMX: thioredoxin, calnexin: protein chaperone, Acsl4: enzyme in steroidogenesis, PTDSS1/2: formation of PS from PC/PE, Mfn2: MITO fusion, DRP1: Mito fission, Atg14, autophagy receptor, Ero1α: oxidoreductase, IP3R, Ca^2+^ channel, MAVS: antiviral signaling	Cui et al., 1993; Stone et al., 2009; Lynes et al., 2012
	VAPB-PTPIP51 tether	Physical tether, may be involved in Ca^2+^ homeostasis	VAPB: ER/MAM protein plays role in UPR, PTPIP51: MITO protein, various functions		Stoica et al., 2014
	Fis1-Bap31 tether	Recruitment of procaspase-8 to MAM leading to induction of apoptosis	Fis1: MITO/PO TA protein involved in MITO/PO fission, Bap31: ER protein involved in quality control		Iwasawa et al., 2011
MITO-LD	Periphilin 5 tether	Physical and metabolic linkage	Periphilin 5: LD-associated scaffold protein		Wang et al., 2011

As an introduction to the Frontiers research topic on “Molecular mechanisms and physiological significance of organelle interactions and cooperation” this review aims at providing a general and timely overview of the new and fascinating mechanisms which convey the cellular plasticity required to react to metabolic and environmental changes in a spatial and temporal manner. We address processes of organelle interaction with a particular focus on membrane contact sites emerging at the cross roads of organelle research and intracellular signaling. In addition, we highlight novel findings on the functional aspects of organelle interaction with a special emphasis on mitochondria and peroxisomes. We particularly focus on organelle interplay in mammals but where appropriate also refer to recent discoveries in plants and fungi.

## Connections between the ER and the plasma membrane

In striated muscle cells a close apposition between peripheral ER and the plasma membrane, now well known as the T-tubule system required for excitation-contraction coupling, was reported as early as the 1950's by the pioneers of cell biological research, Keith Porter and George Palade (Porter and Palade, 1957). Originally regarded as a specialization only found in muscle cells it has in the meanwhile become obvious, that specialized juxtaposed membrane stretches between the ER and the plasma membrane are ubiquitously distributed among eukaryotic cells (Stefan et al., 2013) (Figure 1). While the classical secretory pathway or endosomal trafficking between the ER and the plasma membrane involves the passage of further intermittent organelle structures, the so-called “plasma membrane-associated membrane of the ER” (PAM) represents a direct link between both subcellular compartments (Figure 1). Linked to the function of the peripheral sarcoplasmic reticulum, one of the specializations of the PAM comprises the control of Ca^2+^ dynamics between the extracellular space and the ER, which is the dominant Ca^2+^ storage compartment of the cell. In this respect the plasma membrane of T-tubules is enriched in voltage gated ion channels which activate juxtaposed ryanodine receptors in the PAM to elicit Ca^2+^ into the cytosol (Endo, 2009) (Figure 2A). Both membranes are interconnected by junctophilins, integral membrane proteins of the ER in excitable cells (Figure 2A). Junctophilins stabilize association between the plasma membrane and the ER at junctional complexes by binding to phosphatidylinositol phosphate (PIP) lipids at the cytoplasmic side of the plasma membrane (Takeshima et al., 2015). A more commonly distributed protein assembly found in less specialized cells is the “store-operated calcium entry pathway” (SOCE). This is composed of the ER Ca^2+^ sensor STIM (stromal-interacting molecule), which interacts with the plasma membrane Ca^2+^ channel Orai1 at ER/plasma membrane contact sites in order to replenish ER Ca^2+^ concentrations (Liou et al., 2005) (Figure 2A). Again this process involves the binding of PIP lipids at the plasma membrane by the ER resident STIM protein (Park et al., 2009). Opening of Orai1 channels leads to focally elevated Ca^2+^ concentration at the cytosolic face of the PAM facilitating its uptake by “ER sarcoplasmic/endoplasmic reticulum calcium ATPase” channels (SERCA).

Interestingly, SOCE assemblies have been recently described for the spine apparatus—a stack of smooth ER found in the necks of dendritic spines of principal cortical and hippocampal neurons (Korkotian et al., 2014). An essential component of the spine apparatus is the actin-associated protein synaptopodin (Deller et al., 2000a). Functional studies indicate that the spine apparatus acts as a dynamic intracellular calcium store (Vlachos et al., 2009), involved in regulation of homeostatic synaptic plasticity and memory (Deller et al., 2000a; Vlachos et al., 2013; Korkotian et al., 2014). In line with such a function, characteristic Ca^2+^ ryanodine and inositol tris-phosphate 3-receptors (IP3R) have been described in the ER of dendritic spines (Satoh et al., 1990) (Figure 2A). Thus, plasma membrane/ER associations may act to regulate the Ca^2+^ concentrations in the spine apparatus in order to dynamically control postsynaptic signal transmission. A putative axonal homolog of the SA is comprised of the so-called cisternal organelle which is specifically localized in the axon initial segment (AIS) (Deller et al., 2000b). Structurally, the cisternal organelle is comprised of stacks of smooth ER frequently found in apposition to the AIS plasma membrane. Similar to the SA, synaptopodin is also an essential component for the cisternal organelle (Bas Orth et al., 2007). Additional proteins characteristic for the PAM, including IP3R channels and SERCA pumps, have been found in the cisternal organelle (Benedeczky et al., 1994; Sánchez-Ponce et al., 2011). Although, the precise function of the cisternal organelle is still unknown, it may act as a distinct axonal ER Ca^2+^ storage compartment which mediates calcium-dependent signal transmission in cooperation with apposed ion channels in the plasma membrane (King et al., 2014). In this respect both the spine apparatus and the cisternal organelle may represent neuron-specific specialized PAM regions which create the Ca^2+^ microenvironments required in specific subcellular compartments of highly polarized neurons.

Comparable to the specialization of the mitochondria associated membrane of the ER (MAM), the PAM is also supposed to be involved in the transfer of lipids to the opposing plasma membrane. While the MAM delivers phosphatidylserine to mitochondria [see Section The Mitochondria-associated Membrane of the ER (MAM)], the PAM is involved in the transport of sterol compounds between the ER and the plasma membrane (Toulmay and Prinz, 2011) (Figure 1). In yeast, oxysterol-binding protein (OSBP)-related proteins (ORPs) have been proposed as shuttles between apposed ER/plasma membrane sites (Schulz et al., 2009) and deletion of all ORPs in a yeast strain has been shown to decrease sterol exchange significantly (Beh et al., 2001). A subset of yeast ORPs possess Pleckstrin homology domains and a motif containing two phenylalanine residues in an acidic tract (FFAT), which bind PIPs of the plasma membrane and Vamp-associated proteins of the ER membrane (VAP), respectively (Roy and Levine, 2004; Loewen and Levine, 2005). Both structures ensure that the proteins target to ER/plasma membrane contacts thereby generating focal lipid exchange sites. Sterol lipid exchange between the opposing membranes appears to function in both directions and implies a complex lipid sensing system which is still not completely understood (see Toulmay and Prinz, 2011; Stefan et al., 2013 for detailed information). In this context, yeast ORPs (Osh proteins) appear to fulfill a role beyond mere sterol shuttles, also acting as lipid sensors, transmitting signals to upstream regulators. The Osh proteins localize to ER/PM contacts after phosphatidylinositol 4-phosphate (PI4P) binding and interaction with the VAP Scs2 (Stefan et al., 2011). PI4P binding to Osh prevents sterol loading at the plasma membrane, which exhibits high PI4P levels (Stefan et al., 2013). In this context, a further interaction of Osh with the PIP phosphatase Sac1 could act as a reciprocal regulation circuit facilitating the extraction of sterols from the plasma membrane by reduction of PI4P levels (Stefan et al., 2011).

ORPs (like VAPs) are conserved in higher eukaryotes (Ngo et al., 2010) and a role for mammalian ORPs in the trafficking of sterols from the plasma membrane to the ER and lipid droplets has been described recently (Jansen et al., 2011). Thus, comparable regulation mechanisms may exist in higher eukaryotes.

Three protein families have been recently identified to physically link the ER with the plasma membrane at contact sites in yeast: tricalbins, VAPs, and Ist2 (related to mammalian TMEM16 ion channels) (Manford et al., 2012). Knockout of all tethering proteins not only disrupted PIP signaling but also caused a constitutive activation of the ER unfolded protein response (UPR). Recently, the three mammalian tricalbin homologs, the extended synaptotagmins E-Syt1-3 have been functionally characterized (Figure 2A). All three were shown to tether the ER to the plasma membrane by binding to PI(4,5)P_2_ emphasizing the importance of ER/PM contact sites across species (Giordano et al., 2013; Fernández-Busnadiego et al., 2015). Thus, ER/PM contact sites appear to be required for maintenance of ER physiology, which imply that they are integrated into signaling pathways which cope with the general regulation of cellular homeostasis.

Remarkably, in addition to the ER/plasma membrane contact sites described above, mitochondria are also frequently observed in several cell types in proximity to the cellular surface; e.g., in HeLA cells up to 10% of mitochondria are found beneath the plasma membrane (Frieden et al., 2005). In contrast to the ER, however, mitochondria do not seem to be frequently directly connected to the plasma membrane but appear to be linked via discrete ER-cisternae (Csordás et al., 2010) or filamentous adherence plaques associated to additional vesicular structures along neuronal synapses (Spirou et al., 1998; Rowland et al., 2000). Functionally, these structures may distribute calcium waves from the extracellular space to these calcium storing organelles. In this respect, neuronal mitochondria show specific vulnerability to the elevated excitatory influx of Ca^2+^, which can eventually impair mitochondrial functions (Connolly and Prehn, 2015). Mitochondria from individuals with mutations in the Surfeit locus protein 1 (Surf1) gene show only partially assembled cytochrome C oxidase complexes (3rd complex of the electron transport chain) resulting in the lethal Leigh syndrome in humans (Zhu et al., 1998). Remarkably, neurons of Surf1 KO mice, which show no Leigh-like phenotype, are refractory against glutamate induced Ca^2+^ stress and exhibit an increased life span and enhanced cognitive abilities (Dell'agnello et al., 2007; Lin et al., 2013). Interestingly, the lack of Surf1 leads to decreased Ca^2+^ influx into mitochondria in response to glutamate-induced excitotoxicity. The authors speculated that the reduced buffering capacity of Surf1 KO mitochondria could determine the saturation of the Ca^2+^ microdomains in the contact sites between mitochondria and the plasma membrane or the ER, thereby promoting the feedback closure of their Ca^2+^ channels (Dell'agnello et al., 2007). Thus, distinct molecular changes in the regulatory organelle framework underneath the neuronal plasma membrane appear to have a direct impact on general neuronal physiology and survival demonstrating the functional significance of organelle contact sites.

## The mitochondria-associated membrane of the ER (MAM)

The increasing application of the transmission electron microscope in the field of cell biology during the 1960s and 1970s already revealed that mitochondria and the ER are often found in close proximity to each other (Copeland and Dalton, 1959; Ruby et al., 1969; Franke and Kartenbeck, 1971; Morré et al., 1971). Co-sedimentation experiments using density gradients further implied that both organelles are indeed physically associated (Pickett et al., 1980; Montisano et al., 1982). In 1990, however, J. Vance discovered that the microsomes co-sedimenting with mitochondria represent a specialized cellular subcompartment and proposed the name “mitochondria associated membrane of the endoplasmic reticulum” (MAM) (Vance, 1990) (Figure 1). In the decades after this ground-breaking discovery our understanding of the functional significance of the MAM has greatly advanced revealing that this special ER compartment communicates with mitochondria in order to fulfill a plethora of functions associated with, among others, lipid metabolism and Ca^2+^ signaling but also the regulation of mitochondrial maintenance and programmed cell death/cell survival reflecting different levels of complexity (Raturi and Simmen, 2013; Vance, 2014; van Vliet et al., 2014). As these diverse functions imply, it is still not clear if there is one single MAM compartment or if there are several MAMs equipped with a specialized sub-proteome in order to fulfill different functions. The protein assembly found at the MAM is a subset of *bona fide* ER proteins, which are, however, enriched if compared to classic smooth or rough ER fractions. Thus, the enzymatic activities found at the MAM can be also found at other ER sites, but seem to be focused at this specific location. To date only one protein has been described as specific to the MAM—the phosphatidylethanolamine-N-methyltransferase-2 (Cui et al., 1993), which appears to be only expressed in liver (Cui et al., 1995). Proteomic approaches to define the MAM in different tissues led to the identification of approximately 1000 proteins each (Poston et al., 2013; Horner et al., 2015). However, the overlap between proteins identified in different tissues using different approaches is far lower. A significant number of these identifications may arise from contaminating microsomes and mitochondria, which cannot be entirely separated from the MAM fraction. Thus, to define a specific MAM proteome, sophisticated isolation strategies combined with quantitative mass spectrometry approaches are required in the future. Nevertheless, to date a considerable number of proteins is generally accepted to be significantly enriched in the MAM and can be used as marker proteins for this subcompartment (Vance, 2014; van Vliet et al., 2014). Since the MAM is continuous with the remaining ER it is also pertinent to discuss the mechanisms which lead to enrichment of specific proteins in this membrane subcompartment. Commonly, conserved amino acid stretches target specific proteins to their designated compartment, a mechanism which was reported for the MAM-enriched transmembrane protein acyl-CoA:diacylglycerol acyltransferase 2 (DGAT2) (Stone et al., 2009). Corresponding targeting sequences have not yet been confirmed for other MAM proteins but cysteine palmitoylation was recently reported to be required for the sorting of the two MAM-enriched membrane proteins of the thioredoxin family, TMX and calnexin (Lynes et al., 2012). However, there is currently no consensus on a *bona fide* sorting signal for the different types of MAM protein constituents. Other targeting information as well as the lipid membrane environment may ensure that individual proteins are retained in the MAM or even enriched in specific raft-like subdomains of the compartment.

A closer look at the group of enriched proteins, which include, amongst others, long-chain acyl-CoA synthetase-4, phosphatidylserine synthase-1 and -2, mitofusin 2 (MFN2), dynamin-related protein 1 (DRP1), calnexin, autophagy-related protein 14 (ATG14), the oxidoreductase Ero1α and IP3R, reveals that the MAM is a multifunctional compartment which is involved in several metabolic but also regulatory pathways of the cell. In this respect, the MAM is currently supposed to be involved in the processes of (1) phospholipid synthesis and transfer, (2) calcium signaling, (3) mitochondrial fission, (4) mitophagy, (5) ER-stress response, (6) regulation of apoptosis, and (7) inflammatory/antiviral responses (Figure 1), which will be described in more detail in the following paragraph.

Historically, the first function associated with the MAM was its contribution to lipid metabolism (Vance, 1990). The production of phospholipids in order to supply the remaining endomembrane system of the cell is a well-known task of the ER. After synthesis, the phospholipids can be transferred to their destinations by vesicle-mediated transport. However, not all subcellular compartments—e.g., mitochondria and peroxisomes—are supposed to receive phospholipids via such a process, but may rely on a direct transfer between juxtaposed membranes (Voelker, 2009; Prinz, 2010; Schlattner et al., 2014). Mitochondrial membranes are characterized by a high content of phosphatidylethanolamine (PE). PE can be synthesized at the site of the inner mitochondrial membrane from phosphatidylserine (PS) via the PS decarboxylation pathway (Shiao et al., 1995; Birner et al., 2001). PS, however, is synthesized and supplied by the ER (Vance, 1991). Consequently, the MAM, as a site of close apposition between the ER and mitochondria, was found to be strongly enriched in the two PS synthases 1 and 2 (Stone and Vance, 2000). There is strong evidence that PS synthesized at the MAM is subsequently channeled to the mitochondrial inner membrane for further processing into PE, which can be subsequently exported back to the ER or to other subcellular compartments (Vance, 2014). Besides PE, other mitochondrial membrane lipids like phosphatidylcholine or cardiolipin are at least partially supplied in the form of precursor molecules to mitochondria by the ER (Vance, 2014). Since a significant number of lipid-metabolizing enzymes are enriched at the MAM, it is likely that further lipids are transferred between mitochondria and this specialized ER subcompartment (Raturi and Simmen, 2013).

Both, the ER and mitochondria are important intracellular calcium stores and cyclical calcium exchange between both organelles is crucial for cell life and death (Raturi and Simmen, 2013; Marchi et al., 2014). However, Ca^2+^ concentrations of approximately 1 mM inside the ER (de la Fuente et al., 2013) by far exceed those in mitochondria, which are highly dynamic and react to even small Ca^2+^ changes in the cytosol (Giacomello et al., 2007). Calcium ions in mitochondria are required to regulate mitochondrial energy homeostasis by activating the rate limiting enzymes of the Krebs cycle. Moreover, Ca^2+^ is involved in the regulation of mitochondrial motility and apoptosis (Giacomello et al., 2007; Rowland and Voeltz, 2012). Ca^2+^ uptake by mitochondria is electrochemically driven by the electron potential across the inner mitochondrial membrane and facilitated by the mitochondrial low affinity calcium uniporter MCU (Baughman et al., 2011; De Stefani et al., 2011; Chaudhuri et al., 2013). To still allow rapid and highly dynamic Ca^2+^ changes in mitochondria, the close proximity between the MAM and mitochondria creates locally elevated cytosolic Ca^2+^ concentrations (Rizzuto et al., 1998). For this reason the MAM is highly enriched in inositol-1,4,5-tris-phosphate sensitive Ca^2+^ channels (IP3R) which release calcium into the local surrounding cytosol in response to IP3 signaling (Rizzuto et al., 1993; Hayashi et al., 2009) (Figure 2A). Indeed, changes in the distance between the MAM and mitochondrial membranes lead to alterations in the efficiency of Ca^2+^ transfer (Csordás et al., 2010). Moreover, IP3R activity is inhibited by low and very high cytosolic Ca^2+^ concentrations in an autoregulative system (Bezprozvanny et al., 1991). Creating a reciprocal cycle, Ca^2+^ ions can also be released from mitochondria via the Na^+^/Ca^2+^ exchanger NCLX (Palty et al., 2010) and taken up by the calcium pumps of the SERCA family, which may also be concentrated at the MAM (Lynes et al., 2012). To interfere with mitochondrial energy homeostasis, MAM Ca^2+^ release is reciprocally coupled to cytosolic ATP concentration. Specifically, IP3R3 channel activity has been shown to closely depend on free ATP concentrations in the surrounding cytosol (Mak et al., 1999). In addition, IP3R activity is further modulated by numerous control systems. For example, phosphorylation by cAMP-dependent protein kinase (PKA) promotes IP3R1 activity, whereas protein phosphatases 1 and 2A have an inhibitory effect (DeSouza et al., 2002). Moreover, IP3R activity is modulated by further regulatory proteins like the sigma-1 receptor, promyelocytic leukemia (PML) tumor suppressor protein or GRP75/VDAC1 (see Raturi and Simmen, 2013; Marchi et al., 2014 for details) (Figure 2A).

A deregulation of cellular calcium signaling is supposed to be involved in the development of insulin resistance in type 2 diabetes (Guerrero-Hernandez and Verkhratsky, 2014). IP3R calcium release activities were reported to be influenced by an interaction with the GRP75/VDAC1 complex at the MAM (Szabadkai et al., 2006). Interestingly, disruption of MAM integrity and correspondent VDAC1/IP3R1 and Grp75/IP3R1 interactions are associated with altered insulin signaling in mouse and human primary hepatocytes (Tubbs et al., 2014). Likewise, the authors observed that ER—mitochondria contact sites are decreased in established diabetic mouse models. An induction of ER-mitochondria contact sites by pharmacologic treatment or overexpression of the mitochondrial MAM protein cyclophilin D, however, partially restored insulin sensitivity in the mice. Thus, the MAMs role in Ca^2+^-mediated organelle communication does not appear to be restricted to a direct regulation of mitochondrial physiology but may represent a signaling hub, which interferes with higher level networks controlling cellular energy homeostasis.

Considering its role in the regulation of energy homeostasis, it is not surprising that the MAM is also involved in the complex signaling network controlling cell fate decision. Generally, the ER responds to cellular stress, paralleled by accumulation of unfolded protein, with a signal transduction mechanism called “unfolded protein response” (UPR). In this process the ER stops protein translation and activates chaperones assisting protein folding (Schröder and Kaufman, 2005). Chaperone-mediated protein folding is a massively energy demanding process. To maximize ATP production under ER-stress, cells exhibit an increasing number of ER/mitochondrial contact sites leading to elevated mitochondrial Ca^2+^ concentrations and thus higher oxidative ADP-phosphorylation rates (Bravo et al., 2012). However, if the UPR is not able to reduce cellular stress, lethal signaling pathways will be activated finally triggering apoptosis. In such a situation truncated isoforms of SERCA1 localizing to the MAM were reported to be upregulated. This process leads to an increase in ER/mitochondria contact sites, elevated Ca^2+^ leakage and inhibition of mitochondrial movement, thereby causing mitochondrial Ca^2+^ overload which triggers apoptosis (Chami et al., 2008).

Mitochondria are not a static cellular compartment but constantly change their morphology by fusion and fission. Fission of mitochondria is mediated by cytosolic Drp1 which is recruited to mitochondrial constriction sites by several membrane proteins like Fis1, MiD49/MiD51, or Mff (Lee and Yoon, 2014) (see Section Interplay between Peroxisomes and Mitochondria). ER tubules have been found to wrap around mitochondria marking sites of fission by inducing actin assembly at these ER-mitochondria contacts (Friedman et al., 2011; Korobova et al., 2013). These events seem to precede Drp1-induced fission by preformation of a constriction site to which Drp1 is subsequently recruited (Friedman et al., 2011). Indeed, Drp1 was found to colocalize in significant amounts with these ER/mitochondria contacts (Friedman et al., 2011). In yeast the “ER mitochondria encounter structure” (ERMES), a multiprotein complex, has been described as a tethering structure participating, amongst other functions, in lipid transfer and mitochondrial fission (Murley et al., 2013) (Figure 2C). With the conserved “ER membrane protein complex” (EMC), a second tethering complex involved in lipid transport has been described in yeast recently (Lahiri et al., 2014). In higher eukaryotes a direct ERMES homolog has not been identified, whereas candidates for EMC homologs exist but are yet not functionally characterized. Very recently, syntaxin17 was reported to reside at ER/mitochondria contacts and to promote mitochondrial fission by participating in Drp1 assembly at the mitochondrial constriction site in mammalian cells (Arasaki et al., 2015). Mitochondrial fusion is mediated by the dynamin-related proteins Mfn1/2 or Opa1 found at the outer and inner mitochondrial membrane, respectively (Lee and Yoon, 2014). Mfn2 is also a *bona fide* constituent of the MAM, generally supposed to physically tether ER/mitochondria contact sites by interacting with Mfn2 or Mfn1 of the outer mitochondrial membrane (Figure 2A). However, this view has recently been questioned (Cosson et al., 2012; Filadi et al., 2015). In contrast to the general view, the authors come to the conclusion that Mfn2 acts as a negative regulator of organelle apposition (Filadi et al., 2015). In addition to Mfn2, VAPB of the MAM has been recently described to interact with the outer mitochondrial membrane protein PTPIP51 representing an additional physical linker pair between both organelles (Stoica et al., 2014) (Figure 2A). However, the tethering function of Mfn2 at the contact sites between ER and mitochondria appears to be independent from its role in mitochondrial fusion events. Thus, whilst there is already considerable knowledge on the architecture of the MAM, its role in regulation of mitochondrial fission and fusion remains fragmentary. Nevertheless, there is clear evidence that the mitochondrial dynamics are crucial for the regulation of metabolic homeostasis and cell survival (Ni et al., 2015). Mitochondrial elongation rescues mitochondria from autophagy whereas damaged mitochondria appear to lose their fusion capacity preventing their incorporation into the healthy mitochondrial network (Twig et al., 2008). Besides its role as a mediator of mitochondrial fission, the MAM also seems to be more directly involved in the process of mitophagy. There is increasing evidence that the ER supplies membrane material for the formation of autophagosomes (Tooze and Yoshimori, 2010). Interestingly, the pre-autophagosomal protein Atg14 relocalizes from a homogenous ER distribution to the MAM during autophagy-inducing starvation conditions (Hamasaki et al., 2013). Likewise after starvation Atg5 accumulated at ER/mitochondria contact sites. In contrast, disruption of ER/mitochondrial contacts by Mfn2 or PACS2 knockdown attenuated the formation of autophagosomes, implying a role for the MAM in autophagosome formation (Figure 2B). Thus, the MAM may act as a direct linker between phagosome formation and mitochondrial fragmentation, thereby regulating mitochondrial homeostasis. As described above the interaction of the MAM and mitochondria on different mechanistic levels is involved in determining cell survival or death, significantly contributing to the regulation of apoptosis. The communication systems involved in apoptosis described so far predominantly transmit signals from the MAM to mitochondria. A sophisticated regulation system, however, involves feedback loops between communicating cellular entities. In this respect, mitochondrial Fis1 was recently reported to interact with ER Bap31 in order to recruit procaspase-8 to the MAM facilitating its activation into caspase-8 (Iwasawa et al., 2011) (Figure 2A). Subsequently, Ca^2+^ emission from the MAM further elevates mitochondrial Ca^2+^ concentration stimulating the induction of apoptosis.

Further signaling networks, in which mitochondria and the ER were reported to cooperate at the MAM are involved in the activation of the antiviral innate immune response (Marchi et al., 2014; van Vliet et al., 2014). Cytosolic pathogen recognition receptors RIG-I are able to detect cytosolic foreign RNA and subsequently induce the production of type I interferons and proinflammatory cytokines (Sumpter et al., 2005). To this end RIG-I receptors assemble in a multiprotein complex by docking to mitochondria antiviral signaling protein (MAVS)—an adaptor protein located at the outer membrane of mitochondria and peroxisomes (Belgnaoui et al., 2011) (see Section Interplay between Peroxisomes and Mitochondria). In a recent publication the MAVS were shown to reside on the MAM, where they appear in close proximity to peroxisomal and mitochondrial MAVS during viral infection (Horner et al., 2011). This organelle connecting assembly was suggested to act as signaling hub for the regulation of mitochondrial and peroxisomal innate immune responses after viral infection (Horner et al., 2011). In a subsequent publication the authors further reported that the MAM proteome dynamically changes after virus infection in particular increasing the amounts of individual MAVS interacting proteins (Horner et al., 2015). Evaluating their findings, the authors speculated that the MAM may be used to coordinate mitochondrial and peroxisomal metabolism according to the requirements during virus infection.

The Nod-like receptor NLRP3-inflammosome is a large multiprotein complex serving as a platform mediating the activation of interleukins IL1β and IL18 and contributing to innate immunity (Schroder and Tschopp, 2010; Gurung et al., 2015). To this end the NLRP3 senses pathogen- and danger-associated molecular patterns which activate the assembly of the inflammosome. In this respect, signals for mitochondrial dysfunction like ROS or elevated Ca^2+^ efflux stimulate inflammosome assembly (Gurung et al., 2015). Inactive NLRP3 was reported to localize to the ER, but upon inflammosome activation redistributes to ER-mitochondrial clusters comprising MAM sites (Zhou et al., 2011). These events occur in response to elevated mitochondrial ROS production after inhibition of mitochondrial autophagy. Interestingly, knockdown of VDAC1, which promotes mitochondrial Ca^2+^ uptake at the MAM, thus elevating mitochondrial ATP production, significantly reduced inflammosome activation. In this respect, inflammosome formation at the MAM may be a reaction to elevated ROS production during mitochondrial ATP production.

The intriguing diversity of functions associated with the MAM described above vividly illustrates how the cell connects the metabolic control of cellular functions to control circuits of higher order and complexity which finally contribute to the decision of cellular survival and death. In this respect, the findings that the MAM cooperatively interconnects peroxisomal and mitochondrial MAVS signaling (Horner et al., 2011) (see Section Interplay between Peroxisomes and Mitochondria) further directs our view on organellar cooperation to cross-compartment signaling networks which may integrate cellular homeostasis and dysfunction in different locations of the cell.

## The Peroxisome-ER connection

The intricate relationship between the ER and peroxisomes (Figure 1) includes cooperation in various metabolic pathways, for example the biosynthesis of ether-phospholipids (e.g., myelin sheath lipids), which starts in peroxisomes and is completed in the ER, the formation of GPI-anchored proteins in the ER, and the production of polyunsaturated fatty acids (e.g., docosahexaenoic acid) (for a detailed review see Schrader et al., 2013). It is now clear that the ER also has a role to play in the generation of peroxisomes as well as regulation of their function. Study of this relationship began with ultrastructural studies in the 1960's which demonstrated a close proximity between the smooth ER and peroxisomes (Novikoff and Shin, 1964; Novikoff and Novikoff, 1972; Reddy and Svoboda, 1972, 1973). These early images show peroxisomes entwined and engulfed by the tubules of the ER, suggesting an intimate, physical interaction (which may not even leave sufficient space for vesicle-based interaction). Indeed, in those TEM images both organelles appear to be interconnected by electron-dense intermembrane cross-bridges, spanning a distance between 10 and 15 nm (Kartenbeck and Franke, 1974; Zaar et al., 1987), which resemble the ultrastuctural appearance of known organellar contact sites, like the association between MAM and the outer mitochondrial membrane. Importantly, the electron-dense cross-bridges and attached ER tubules could even be visualized, and biochemically verified, in isolated peroxisome fractions (Zaar et al., 1987). Despite this clear and long-held evidence for a specialized ER-peroxisome contact site, its protein composition and physiological function remain obscure but may be broadly associated to two cellular processes: (1) the biogenesis of peroxisomes as derivatives from the ER or (2) the exchange of metabolites from shared biochemical pathways, for example the ether phospholipid biosynthesis.

Confidence in the level of this intimacy, with regards to the ER as the site of peroxisome production has fluctuated over the years. Over 40 years ago Christian De Duve suggested that it was “almost textbook knowledge” that peroxisomes were derived from the ER and that peroxisomal proteins were delivered intraluminally via ER channels (De Duve, 1973). This view was, however, subsequently replaced by the growth and division model of peroxisome biogenesis established by Fujiki and Lazarow (Fujiki and Lazarow, 1985). This model proposed that, although the phospholipids required to form the peroxisome membrane could be provided by the ER, peroxisomal proteins were synthesized on cytoplasmic ribosomes and delivered directly to peroxisomes. There is general agreement that this applies to peroxisomal matrix proteins, whereas delivery of peroxisomal membrane proteins (PMPs) became a matter of ongoing debate.

Over the years a wide variety of evidence has been presented in support of both models and there has been considerable debate as to which mechanism predominates in wild type cells (Hoepfner et al., 2005; Kim et al., 2006; Motley and Hettema, 2007; Nagotu et al., 2008; Delille et al., 2010; Rucktäschel et al., 2010; Van der Zand and Reggiori, 2012). Much of the debate has stemmed from the observation that cells lacking, or carrying mutations in, the peroxisome biogenesis factor Pex3 do not contain peroxisomes (Baerends et al., 1996; Muntau et al., 2000). Pex3 is a membrane protein which, along with its cytoplasmic partner Pex19, forms an import complex required for insertion of peroxisomal membrane proteins (Götte et al., 1998; Rottensteiner et al., 2004). When Pex3 is re-introduced into Pex3 deficient cells, the protein was observed to route first to the ER and then be released in pre-peroxisomal vesicle structures, which were then supplied with PMPs from the ER (Van der Zand et al., 2010; Van der Zand and Reggiori, 2012). This concept was questioned by a recent ultrastructural study which demonstrated that such pre-peroxisomal structures are already present in cells lacking Pex3 (Knoops et al., 2014). The authors suggested that the ER-localization of re-introduced Pex3, and other proteins, could be due to limitations in the resolution of fluorescence microscopy.

Further data in support of a model in which PMPs transit via the ER comes from studies investigating co-translation insertion at the ER membrane. An early study in yeast suggested that PMP50 was synthesized on ER-associated ribosomes (Bodnar and Rachubinski, 1991). This was supported by a more recent global study which investigated the extent of co-translational delivery of proteins to the ER and found a clear enrichment of genes coding for PMPs at ER-anchored ribosomes in yeast and, to a lesser extent, in mammals (Jan et al., 2014). Jan et al. interpreted this finding to show that PMPs are translated at the ER membrane and are presumably inserted into the ER before being delivered to peroxisomes. An exception to this are tail-anchored membrane proteins which are translated on cytoplasmic ribosomes before being delivered to the appropriate organelle (Borgese and Fasana, 2011) and appear to be targeted by species-specific systems. Accordingly yeast peroxisomal tail-anchored proteins go either direct, or via the ER using the “Guided Entry of Tail-anchored Proteins” (GET) system (Mariappan et al., 2010) but mammalian tail-anchored proteins are delivered directly to peroxisomes (Chen et al., 2014; Kim and Hettema, 2015).

Overall there are still some aspects of peroxisome biogenesis which require clarification but the most recent data supports a growth and division model with a role for the ER (dependent on conditions and species) in delivery of phospholipids and some specific PMPs, such as Pex3.

Having established that at least a portion of PMPs can be delivered by the ER another unresolved issue is the mechanism of transport of such proteins, as well as the essential phospholipids required for the peroxisomal membrane. Vesicular transport of PMPs has been demonstrated in an *in vitro* cell-free system (Agrawal et al., 2011) and may involve the Sec16B protein in mammalian cells (Yonekawa et al., 2011), whilst non-vesicular mechanisms have also been reported to exist (Lam et al., 2010). Removal of Sec16B in mammalian cells results in peroxisome elongation, disruption of ER exit sites and redistribution of Pex16 from peroxisomes to the ER (Yonekawa et al., 2011). Based on these observations Yonekawa and colleagues speculated that Sec16B is involved in forming Pex16-containing vesicles in a peroxisome-like domain of the ER. A recent report also highlighted the potential importance of Pex16 in ER-peroxisomal trafficking (Hua et al., 2015). However, the validity and scope of such a mechanism, and the precise role for Sec16B in this process remains unclear.

Although it is generally accepted that the phospholipids generating peroxisomal membranes come from the ER there are relatively few studies on this process. One such study in yeast, supporting a non-vesicular mechanism, used an engineered strain in which the PTS1 enzyme responsible for the decarboxylation of phosphatidylserine (PS) to phosphatidylethanolamine (PE) was artificially targeted to peroxisomes (Raychaudhuri and Prinz, 2008). In a strain where the endogenous PTS1 genes were removed this allowed monitoring of lipid transfer by measuring the conversion of radiolabelled PS (generated exclusively in the ER) to PE which could now only occur in peroxisomes in this system. The authors found that PS transfer to peroxisomes occurred under normal conditions and also under conditions where vesicular transport was compromised.

Despite a wealth of evidence suggesting a direct, physical interaction between peroxisomes and the ER, understanding of the molecular basis of such contacts is limited. So far there are only a small number of studies reporting a physical tether between the ER and peroxisomes analogous to the complexes which anchor the ER to other organelles such as mitochondria, the PM or lysosomes [see Sections Connections between the ER and the Plasma Membrane, The Mitochondria-associated Membrane of the ER (MAM), Interplay between Peroxisomes and Mitochondria, and Lysosomal Interactions and Autophagy for details and (Prinz, 2014) for a comprehensive review on membrane contact sites]. However, by comparison with other ER-anchoring systems it is likely that there are several tethers connecting the ER to peroxisomes (Stefan et al., 2013). So far in yeast two potential tethering complexes have recently been identified. A complex involving Pex30 has been implicated as a facilitator between peroxisomes and the ER along with a tether involving Pex3 and Inp1 (David et al., 2013; Knoblach et al., 2013). The Pex30 anchoring complex is involved in the regulation of peroxisome proliferation and requires the integrity of the ER tubular network. Through interaction between, among others, Pex30 and the ER proteins, Rtn1, Rtn2, and Yop1 an “ER-peroxisome contact site” (or EPCON) is generated to facilitate ER-peroxisome interactions (David et al., 2013). However, a detailed interaction map of this macromolecular complex bridging both organelles remains to be specified. The authors speculate that these EPCONs could represent a platform from which peroxisomes could be formed. The Pex3-Inp1 tethering system is based on Pex3 being resident in both the ER and peroxisomal membrane and Inp1 acting as a molecular hinge interacting directly with both Pex3 proteins. This tether reportedly functions to regulate the maintenance of peroxisome numbers during budding (Knoblach et al., 2013). Knoblach and colleagues postulate that this occurs by the anchoring, via Inp1 and Pex3, of peroxisomes to the cortical ER prior to division. When division is signaled the peroxisomal division machinery assembles (see Section Interplay between Peroxisomes and Mitochondria) leading to a pulling force which elongates the peroxisome, eventually leading to fission. The newly-formed peroxisomal structure can then move from the mother cell and into the bud. There is no homolog of Inp1 in metazoa so the relevance of a similar tether in other systems is unclear and may be specific to budding yeast.

As initially indicated, ER-peroxisome contacts are extensively observed in mammalian cells and likely represent functionally specialized contact sites comparable to the MAM or PAM described above. However, it remains to be determined if these numerous appositions between both organelles predominantly mirror the process of peroxisome biogenesis or if they mainly contribute to several other cellular processes including exchange of metabolites, such as precursors of ether phospholipids, polyunsaturated fatty acids and cholesterol or even regulation of viral defence (see Section Interplay between Peroxisomes and Mitochondria). Thus, their contribution to peroxisome biogenesis is just one aspect of their multiple functions and it will be challenging to unravel their actual function in different experimental models and set ups.

## Interplay between peroxisomes and mitochondria

In recent years, convincing evidence for a close connection between peroxisomes and mitochondria has been obtained (Schrader and Yoon, 2007; Camões et al., 2009; Delille et al., 2009; Schrader et al., 2012, 2015) (Figure 1). Peroxisomes and mitochondria cooperate in cellular lipid metabolism, in particular the breakdown of fatty acids via their organelle-specific β-oxidation pathways and can both act as subcellular source, sink or target of ROS (Schrader and Fahimi, 2006; Wanders and Waterham, 2006; Antonenkov et al., 2010; Ivashchenko et al., 2011; Fransen et al., 2013). Although peroxisomes and mitochondria can be observed in close proximity, e.g., in ultrastructural studies in mammalian cells and can also be co-purified at distinct buoyant densities (Hicks and Fahimi, 1977; Islinger et al., 2006), studies on the molecular background of physical interactions and their physiological importance are scarce (Horner et al., 2011, 2015; Van Bergeijk et al., 2015). Recent studies in yeast localized peroxisomes to specific mitochondrial subdomains such as mitochondria-ER junctions and sites of acetyl-CoA synthesis (Cohen et al., 2014). In line with this, a genome-wide localization study of peroxisome-mitochondria interactions in yeast identified Pex11, a membrane-bound peroxin (peroxisome biogenesis factor) involved in peroxisome division and proliferation, and the mitochondrial ERMES complex (Mattiazzi Usaj et al., 2015) (Figure 2C). The ERMES complex is supposed to provide a tether and to facilitate the exchange of molecules between the ER and mitochondria. In particular, Pex11 was found to physically interact with Mdm34 to establish the contact sites between peroxisomes and mitochondria (Figure 2C). Interestingly, this interaction was only observed in glucose media, but not after induction of peroxisome proliferation by fatty acids in the absence of glucose. The authors speculate that besides its role in elongation and fission of the peroxisomal membrane, Pex11 may also be a sensor of the metabolic state of peroxisomes. Thus, metabolic stimuli may modulate the peroxisome-mitochondrion tether in yeast. Tethering of both organelles may enhance metabolism by reducing the distance for efficient transport of metabolites from one organelle to another. Mammalian cells lack ERMES, and another tethering complex is supposed to perform similar functions in higher eukaryotes.

Tethering might also play a role in the coordinated movement of both organelles, in particular for organelle inheritance. Whereas in budding yeast distinct organelle-specific membrane proteins are involved in the actin-myosin dependent inheritance of peroxisomes and mitochondria (Knoblach and Rachubinski, 2015), in the fission yeast *Schizosaccharomyces pombe* peroxisome movement in association with mitochondria has been reported (Jourdain et al., 2008). Another example is the red algae *Cyanidioschyzon merolae*, which possesses only one peroxisome and one mitochondrion. During coordinated organelle-division the peroxisome interacts with the mitochondrion to partition into the daughter cell (Miyagishima et al., 1999). Note that in budding yeast tethering of peroxisomes and mitochondria to the ER is crucial for organelle retention and inheritance (see Section The Peroxisome-ER connection).

Another interesting twist of the peroxisome-mitochondria connection is the discovery that peroxisomes and mitochondria share key proteins of their division machinery (Schrader et al., 2012), namely the dynamin-related GTPase Drp1/DLP1/(Koch et al., 2003; Li and Gould, 2003), its membrane adaptor proteins Fis1 and Mff (Koch et al., 2005; Gandre-Babbe and Van Der Bliek, 2008; Otera et al., 2010; Koch and Brocard, 2012; Itoyama et al., 2013) as well as GDAP1, a putative GST-transferase (Huber et al., 2013) in mammals. Fis1 and Mff are supposed to recruit the mechanochemical enzyme Drp1 to distinct spot-like division sites at the organelle membrane prior to fission. Sharing of key division components is conserved in mammals, fungi, yeast, and plants (Delille et al., 2009; Schrader et al., 2012), The first patients with defects in different division proteins (e.g., Drp1, Mff, Pex11β) and thus, an abnormal elongated organelle morphology, have been identified underlining the biomedical importance of membrane deformation and fission (Waterham et al., 2007; Ebberink et al., 2011; Ribeiro et al., 2012; Shamseldin et al., 2012; Schrader et al., 2014). Unraveling how a cell is able to timely coordinate the distribution of shared components of the mitochondrial and peroxisomal division machinery in order to meet the requirements of increased organelle-specific proliferation will be a challenging task for future research activities and may involve hitherto undetected networks of organelle cross-talk.

In addition to the key division proteins, the division of mitochondria involves ER-mitochondria contacts (Friedman et al., 2011), and actin filaments (Li et al., 2015). ER tubules were observed to wrap around mitochondria in yeast and mammalian cells, to mark fission sites and to drive mitochondrial constriction (Friedman et al., 2011; Korobova et al., 2013). It is unknown if peroxisomal membrane fission is also ER-assisted. Recent *in vitro* studies using liposomes and recombinant Pex11β imply that membrane constriction may occur unassisted by ER (Yoshida et al., 2015).

The constitutive formation of organelles also requires degradation of faulty or surplus organelles. This is achieved by autophagic processes (pexophagy, mitophagy). The size of the organelle is a critical factor for the efficient engulfment by the sequestering compartment, the phagophore. Organelle fission is critical for the efficient elimination of mitochondria (Gomes and Scorrano, 2013) and peroxisomes (Mao et al., 2014). In *S. cerevisiae*, it was reported that pexophagy-specific fission, mediated either by the dynamin-like GTPases Dnm1 or Vps1, occurred at mitochondria-peroxisome contact sites. The authors suggest that whereas division of mitochondria requires the participation of the ER, the fission of yeast peroxisomes may involve mitochondria (Mao et al., 2014). It should be noted that as both organelles are in intimate contact with the ER (see Section The Peroxisome-ER Connection), potential peroxisome-mitochondria contacts might be indirect and mediated by ER membranes.

Mitochondria, and increasingly also peroxisomes, are now recognized as important signaling nodes in the cell and cooperative functions in anti-viral and redox signaling are emerging (Dixit et al., 2010; Fransen et al., 2013; Odendall and Kagan, 2013; Nordgren and Fransen, 2014). With the discovery of the dual distribution of mitochondrial antiviral signaling protein (MAVS) to both peroxisomes and mitochondria, a novel role for peroxisomes in the innate immune response of the host cell to combat viral and bacterial infections, either alone or in cooperation with mitochondria, was revealed (Dixit et al., 2010; Odendall et al., 2014). MAVS functions as an adaptor protein for retinoic acid-inducible gene 1 protein (RIG-I) and transmits downstream signaling of antiviral immunity. Interestingly, MAVS localizes to mitochondria-associated ER membranes (MAM) and dynamic MAM tethering to mitochondria and peroxisomes is supposed to coordinate MAVS localization to form a signaling synapse between membranes. It could regulate the interaction between positive and negative regulators distributed on different organelles in order to fine-tune the RIG-1 induced innate immune response (Horner et al., 2011) (Figure 1). Proteomic analysis of MAM during RNA virus infection revealed an increased presence of peroxisomal proteins if compared to control cells, supporting physical interactions between peroxisomes and mitochondria (or MAM) during anti-viral response (Horner et al., 2015).

It is becoming increasingly evident that peroxisomes and mitochondria also share an intricate redox-sensitive relationship. Both organelles are crucial for cellular redox homeostasis (Nordgren and Fransen, 2014). Interestingly, disturbances in peroxisomal lipid and ROS metabolism have an impact on the mitochondrial redox balance (Koepke et al., 2008; Ivashchenko et al., 2011; Walton and Pizzitelli, 2012). It is hypothesized that such peroxisomal disturbances can trigger redox-related signaling events that ultimately result in increased mitochondrial stress and the activation of mitochondrial stress pathways (Titorenko and Terlecky, 2011; Beach et al., 2012; Fransen et al., 2013). It is, however, unknown, how those signals are transmitted between peroxisomes and mitochondria. Interorganellar communication may involve diffusion of signaling molecules from one organelle to another, communication via membrane contact sites or vesicular transport. With respect to direct membrane contact, it is tempting to speculate that the MAM may contribute to the transmission of ROS and stress responses from peroxisomes to mitochondria. It should be noted, that loss of peroxisomal biogenesis and metabolism, a hallmark of Zellweger syndrome, is associated with impaired mitochondrial integrity. Recent studies in Zellweger-mouse models revealed impaired mitochondrial respiration, DNA depletion, PGC-1α independent proliferation of mitochondria and perturbed carbohydrate metabolism in peroxisome-deficient hepatocytes (Peeters et al., 2011, 2015). These findings suggest an impact on organelle interplay in Zellweger spectrum patients. Concerning vesicular transport, mitochondria have been reported to generate so called mitochondria-derived vesicles (MDVs) that can transport specific mitochondrial proteins to either peroxisomes or to lysosomes for degradation (Neuspiel et al., 2008; Soubannier et al., 2012). The physiological role for peroxisome-directed MDVs is currently unclear. Peroxisomes may also be able to generate and target vesicles to mitochondria, but experimental evidence for this phenomenon is missing. Finally, live cell imaging of peroxisomes in mammals and plants revealed that peroxisomes can form tubular membrane protrusions, which vividly extend and retract, and are thought to mediate interactions with other peroxisomes and organelles. Very recently, peroxisomal membrane extensions were reported to mediate contact with oil bodies (see Section Lipid Droplets) in the model plant *Arabidopsis thaliana* and to deliver a membrane-bound lipase, required for lipid mobilization during seedling establishment (Thazar-Poulot et al., 2015). Membrane protrusions may also be involved in the transfer of membrane lipids. Remarkably, transient contacts between peroxisomes and lysosomes are thought to mediate transfer of cholesterol from lysosomes to peroxisomes (Chu et al., 2015) (Figure 1). Contacts are mediated by synaptotagmin VII on lysosomes which binds to the lipid PI(4,5)P_2_ at the peroxisomal membrane (Figure 2A). LDL-cholesterol enhances such contacts, whereas peroxisome dysfunction results in cholesterol accumulation in lysosomes (Chu et al., 2015). This cholesterol trafficking blockage may contribute to the pathology of peroxisome disorders. An intriguing idea is that peroxisomes may associate with other organelles and deliver cholesterol to them (Chu et al., 2015). This can be mediated by transient organelle contacts or by membrane protrusions. Interestingly, transient contacts between individual peroxisomes have been reported (Bonekamp et al., 2012). These contacts do not result in the exchange of peroxisomal matrix or membrane proteins, but have been suggested to contribute to the equilibration of the peroxisomal compartment in the cell and might instead mediate the transfer of lipids or cholesterol between peroxisomes for further modification. These exciting novel findings underline the role of peroxisomal membrane dynamics in inter-organelle communication and protein/lipid transport and highlight the clinical relevance of these processes.

## Lipid droplets

Lipid droplets (LDs) are specialized organelles involved in the storage of neutral lipids, mainly triacylglycerols, and sterol esters, for energy and membrane homeostasis. LDs have been found in all eukaryotic and some prokaryotic cells, since lipids are essential for life and the capacity to store lipids confer an evolutionary advantage to the organism. The concept that LDs are simple, inert lipid-storage containers has now been dismissed. Today it is widely accepted that LDs are dynamic organelles which are involved in multiple cellular processes including lipid metabolism, but also protein sequestration/degradation and pathogen replication (Palacpac et al., 2004; Welte, 2007; Sorgi et al., 2009; Vogt et al., 2013). LDs are thought to originate from the ER and grow by fusion through a SNARE-mediated process (Böstrom et al., 2007; Murphy, 2012; Walther and Farese, 2012). They are known to move bi-directionally on microtubules and there is significant evidence showing that LDs dynamically interact with other organelles (Figure 1). LDs have been found in close association with ER, peroxisomes, mitochondria, endosomes, and the plasma membrane (Goodman, 2008; Murphy et al., 2009; Dugail, 2014). Lipid-exchange is likely to be the functional linkage between LDs and ER, peroxisomes and mitochondria. The association between the ER and LDs seems to occur even after budding of the LDs, with permanent contacts between these organelles being reported in different cells types (Blanchette-Mackie et al., 1995; Szymanski et al., 2007). Peroxisomes and mitochondria are frequently found in close association with LDs (Novikoff et al., 1980; Schrader, 2001; Binns et al., 2006; Sturmey et al., 2006; Shaw et al., 2008). Those contacts may link fatty acid supply by lipolysis in LDs with peroxisomal and mitochondrial fatty acid β-oxidation. In addition, exchange of lipids between LDs and peroxisomes or mitochondria may also serve membrane replenishment or storage in LDs. Defects in peroxisomal β-oxidation or absence of peroxisomes have been associated with enlarged LDs (Dirkx et al., 2005; Zhang et al., 2010). Inhibition of lipid mobilization in plants resulted in enlarged LDs and clustering of peroxisomes around them (Brown et al., 2013). Recently, it was reported that fatty acids stored in LDs in well-fed cells travel from LDs into mitochondria when cells are kept under starvation conditions. This transfer was dependent on mitochondrial fusion dynamics and close proximity to LDs (Rambold et al., 2015). Endosomes have also been observed to enwrap LDs, potentially promoting the delivery of LDs to lysosomes allowing for the transfer of cholesterol (Martin and Parton, 2005; Ouimet et al., 2011). Even though the interaction of LDs and other organelles (e.g., endosomes, ER, and vacuole) appears to be regulated by several Rab GTPases (Liu et al., 2007; Murphy et al., 2009; Bouchez et al., 2015), and the fusion events between LDs themselves, or LDs and mitochondria likely involves SNARE-mediated homotypic fusion (Goodman, 2008; Jägerström et al., 2009; Olofsson et al., 2009), the underlying molecular mechanisms remain largely unknown. In this respect, the LD-associated protein perilipin 5 is regarded as a candidate for the physical and metabolic linkage of mitochondria to LDs (Wang et al., 2011), whereas the molecular basis for a peroxisome—LD interaction remains elusive. A remaining question is the contribution of protein-protein and/or protein-phospholipid interactions to LD-organelle contacts. Hemi-fusion-like mechanisms would, however, represent an efficient way with low energy cost to exchange lipids between LDs and other organelles (Murphy et al., 2009; Olofsson et al., 2009).

## Lysosomal interactions and autophagy

The first sign of the existence of an organelle with lytic function, known today as lysosomes, arose from the lab of Christian de Duve in 1949. Later on, the first electron microscopy image of lysosomes was obtained in collaboration between de Duve and Novikoff (Novikoff et al., 1956). Through ultrastructural studies researchers observed that lysosomes show pronounced cellular heterogeneity and individual polymorphism. In these pioneering cell biological studies vacuoles containing various organelles in different stages of degradation were observed in the proximity of the ER and the endosomal-lysosomal compartment (Novikoff, 1959; Novikoff and Essner, 1962). As these membrane-surrounded structures were soon discovered to contain the lysosomal marker enzyme acid phosphatase, de Duve proposed that they may be involved in the constitutive removal of cellular material and named them autophagic vacuoles/autophagosomes (De Duve, 1963). Not much later he already hypothesized that the process of autophagy could represent a tightly regulated process involving an autophagic membrane originating from the ER segregating impaired organelles from the remaining pool and a subsequent fusion with primary lysosomes in order to digest the enclosed material (De Duve and Wattiaux, 1966). Thus, even if the origin of the autophagic membrane is still not resolved, it is quite obvious, that the process of autophagy involves controlled interaction between (1) the segregation membrane and an impaired organelle and (2) the autophagosome and a primary lysosome/endosome. Consequently, autophagy can be regarded as a highly specialized process of organellar interactions organizing cellular maintenance (Figure 1).

Cellular homeostasis can be disturbed due to cellular damage caused by nutrient deprivation, genetic alterations, or aging. To prevent cellular damage, a large array of quality control processes is available to the cell. Autophagy is one such process, consisting of the removal/recycling of cytoplasmic materials (e.g., protein aggregates, lipids, ribosomes, and organelles) by delivering them to the lysosome (Mizushima et al., 2011; Choi et al., 2013). Autophagy can be divided into 3 types: macroautophagy, microautophagy, and chaperone-mediated autophagy (CMA). Macroautophagy is the most understood autophagic process of the three, largely due to the extensive yeast genetic studies which have led to the identification of more than 35 autophagy-related (ATG) genes, along with their corresponding mammalian homologs (Mizushima et al., 2011). In macroautophagy components of the cytoplasm are engulfed by the phagophore (the so-called isolation membrane) leading to the formation of the autophagosome (double-membrane structure) (Mizushima et al., 2011). Maturation of the autophagosome occurs by fusing to endosomes and eventual engulfment by lysosomes, where it is degraded along with the cytoplasmic materials present in it. The autophagosome-lysosome fusion was found to be mediated by the SNARE Syntaxin 17 protein (Itakura et al., 2012). Special types of macroautophagy have been reported depending on the type of organelle; mitochondria (mitophagy), peroxisomes (pexophagy), lipid droplets (lipophagy), ER (reticulophagy), and microbes (xenophagy) (Klionsky et al., 2007).

Peroxisome and mitochondria homeostasis is attained by ensuring equilibrium between organelle biogenesis and degradation. The selective degradation of superfluous or damaged organelles is achieved by either a non-selective or selective autophagic process. During starvation or nutrient deprivation, non-selective autophagy is the predominant process, in order to ensure cell survival by providing essential amino acids and nutrients to the cell. However, under nutrient-rich conditions selective autophagy usually occurs to ensure the removal of damaged or superfluous organelles (Nordgren et al., 2013; Sureshbabu and Bhandari, 2013). In the selective autophagy pathway the specific phagophore membrane required for each form of selective autophagy recognizes the specific cargo prior to delivering it to the vacuole/lysosomal for degradation. The origin of the phagophore membrane still remains controversial. Recent studies have pointed to several organelles as potential membrane source (PM, Golgi, ER, and mitochondria) (Hailey et al., 2010; Mari et al., 2011; Bernard and Klionsky, 2013; Hamasaki et al., 2013). In yeast the mechanism of recognition of specific cargo for both pexophagy and mitophagy is well understood. For methylotrophic yeasts (e.g., *P. pastoris*) the pexophagy receptor is Atg30, which interacts with peroxisomal membrane proteins Pex3, Pex14, and Atg37 (Till et al., 2012; Nazarko, 2014). However, for *S. cerevisiae* and related yeasts the pexophagy receptor is Atg36 and appears to interact solely with Pex3 (Motley et al., 2012). Both Atg30 and Atg36 need to be activated by phosphorylation in order to interact with the scaffold protein Atg11 and the autophagosome via Atg8 (Farré et al., 2013). Surprisingly, Atg30 and Atg36 display no similarities at the amino acid level even though they exhibit similar function (Van der Zand and Reggiori, 2012). In yeast mitophagy the mitochondria outer membrane protein Atg32 was identified as the mitophagy receptor (Kanki et al., 2009; Okamoto et al., 2009). When phosphorylated it interacts with Atg11 and Atg8 on the autophagosome (Farré et al., 2013). Recent reports have shed some light over the signaling events that govern pexophagy/mitophagy, which are still largely unknown. In *S. cerevisiae*, two MAPK kinases, Hog1 and Pbs2, are exclusively required for mitophagy (Mao et al., 2011), whereas the MAPK kinase Slt2 was shown to be required for pexophagy (Manjithaya et al., 2010). Recently the Hrr25 kinase was identified as the responsible kinase for the phosphorylation of Atg19 and Atg36. Hence, enhancing the interactions between these receptors and the mutual adaptor Atg11 (Tanaka et al., 2014). Despite the fact that for methylotrophic yeasts the kinase responsible for phosphorylation of Atg30 is still unknown, a distinct At30-binding domain was recently identified in Pex3 which was important for the phosphorylation of Atg30 and the recruitment of Atg11 by Atg30 (Burnett et al., 2015). Furthermore, it was recently reported that a MAP kinase phosphatase 1 (MKP1) harboring a novel PTS1 (SAL) is targeted to peroxisomes under stress conditions in *Arabidopsis thaliana*. Whether this phosphatase is involved in plant pexophagy is still unknown since the regulatory role of MKP1 was not identified (Kataya et al., 2015).

In contrast to yeast, mechanistic understanding of pexophagy in mammals is more limited. Three pathways have been proposed for degradation of peroxisomes (Figure 2B): (1) p62-mediated detection of an ubiquitinated, unknown peroxisomal membrane protein, followed by autophagosome recruitment via p62 and LC3-II interaction (Kim et al., 2008), (2) direct binding of LC3-II to Pex14, by competing with the binding of Pex5 to Pex14 depending on the nutrient conditions (Hara-Kuge and Fujiki, 2008), (3) binding of NBR1, another adaptor protein like p62, to an ubiquitinated peroxisomal membrane protein or through direct binding to the peroxisomal membrane (Deosaran et al., 2013). This last pathway also includes p62 as another interacting protein, but downstream of the obligate NBR1, supposedly acting as an accessory interaction partner in the tethering complex (Figure 2B).

Also, for mammalian cells a two-step model for priming mitochondria for mitophagy has been proposed: RING-between-RING E3 ubiquitin ligase Parkin dependent or Parkin independent (Ding and Yin, 2012) (Figure 2B). In the Parkin dependent pathway, PINK1 is constitutively cleaved by the mitochondrial protease PARL (Jin et al., 2010). Inactivation of PARL, due to mitochondria membrane depolarization, blocks PINK1 cleavage and access to the inner mitochondrial membrane and subsequently the PINK1 precursor is stabilized at the outer mitochondrial membrane (Meissner et al., 2011). At the outer mitochondrial membrane, PINK1 recruits and activates cytosolic Parkin which then promotes ubiquitination of mitochondria outer membrane proteins (Lazarou et al., 2013; Kane et al., 2014). P62 recognizes ubiquitinated proteins and through its direct interaction with LC3-II recruits autophagosomal membranes to the mitochondria. Parkin and PINK1 have been reported to interact with several other cellular proteins that might be involved in their regulation. For the Parkin independent mechanism, mitophagy is mediated by FUNDC1, Nix, and BNIP3 which interact directly with LC3-II promoting the recruitment of autophagosomes to mitochondria (Ding and Yin, 2012; Jin and Youle, 2012). Interestingly, cardiolipin, a phospholipid of the inner mitochondrial membrane, is transferred to the outer mitochondrial membrane of compromised mitochondria (Chu et al., 2013, 2014). There it can be bound by LC3 via several clusters of basic amino acids on the protein's surface, thereby triggering autophagy. The authors further speculated that cardiolipin peroxidation, resulting from excessive mitochondrial ROS production, could serve to switch between the processes of mitophagy and programmed cell death (Chu et al., 2014). More recently, two other pathways to target mitochondria for mitophagy have been reported. The formation of mitochondria-derived vesicles (MDVs) (Soubannier et al., 2012) (Figure 1), and mitochondria spheroids (Ding et al., 2012) that may delivery mitochondrial components for degradation to the lysosomes and the direct recruitment of p62 via choline dehydrogenase (CHDH) in response to mitochondrial membrane depolarization (Park et al., 2014). In selective autophagic processes, required for degradation of faulty or surplus organelles, the size of the organelle is a critical factor for obtaining efficient engulfment by the autophagosome (Müller and Reichert, 2011; Mao et al., 2014). Peroxisomes and mitochondria fission/fragmentation is a requirement for both selective autophagy processes, and the dynamin-like GTPase DLP1 has been reported to be recruited and activated before either pexophagy or mitophagy occur (Twig and Shirihai, 2011; Mao et al., 2014). However, not all the fragmented organelles are triggered for elimination, indicating that there must be a mechanism that regulates which organelles need to be eliminated. For mitochondria it has been revealed that fission followed by selective fusion of mitochondria and tubular network formation under nutrient deprivation conditions protects mitochondria from mitophagy (Twig et al., 2008; Rambold et al., 2011). In addition, mitophagy is avoided if the membrane potential of the mitochondria is sustained after fission events (Twig et al., 2008). On the other hand, peroxisomes are not able to fuse with one another (Bonekamp et al., 2012) and also do not possess a membrane potential, so the regulatory mechanism must be distinct from the ones available to mitochondria. One hypothesis to discriminate healthy peroxisomes from the ones that need to be degraded might be via asymmetric fission/division of the organelle (Nordgren et al., 2013). A recent study showed that removal of protein aggregates present in the lumen of peroxisomes and its subsequent elimination by autophagy, was achieved by asymmetric peroxisome fission to separate the aggregate from the mother peroxisome (Manivannan et al., 2013). Besides the physical interactions between peroxisomes and autophagosomes, required for pexophagy and subsequent fusion to lysosomes, a very recent study has shown for the first time the existence of lysosomal-peroxisome membrane contacts (LPMC) essential for the cellular trafficking of cholesterol (Chu et al., 2015) (Figure 1). In a well-designed set of experiments the authors showed that the lysosomal Syt7 protein binds peroxisomal PI(4,5)P2 (phospholipid), bridging the organelles and allowing cholesterol to transfer from lysosomes to peroxisomes (Figure 2A). Furthermore, the authors propose a central role for peroxisomes in intracellular cholesterol trafficking and that intracellular cholesterol accumulation may underlie the pathological mechanism of peroxisome disorders (Chu et al., 2015). Thus, lysosomes not only interfere with other subcellular compartments in terms of removal of compromised organelles but appear to be involved also in functional networks which guarantee cellular maintenance.

## Concluding remarks

The current examples for organelle interaction in mammalian cells, as discussed in sections Connections between the ER and the Plasma Membrane, The Mitochondria-associated Membrane of the ER (MAM), The Peroxisome-ER Connection, Interplay between Peroxisomes and Mitochondria, Lipid Droplets, and Lysosomal Interactions and Autophagy, clearly illustrate that subcellular organelles are integrated in cooperating cellular networks. Although intimate physical contacts between organelles were described some time ago, we are just beginning to reveal the key components involved and their physiological importance. A major role of organelle interaction is clearly in metabolite exchange, but exciting new functions in organelle distribution and membrane dynamics have been discovered. Furthermore, increasing evidence points to an important function in signaling and the assembly of dynamic signaling platforms according to cellular requirements. In this respect, higher ordered complexes between more than two organelles may exist as exemplified by antiviral signaling via MAVS involving the ER, mitochondria and peroxisomes. A common principle may be the involvement of structurally similar or overlapping protein complexes for the physical tethering of different organelle membranes. Future studies will reveal if organelle interplay and cooperation is primarily mediated via those hubs, or if indirect mechanisms via the cytosol are more prevalent.

### Conflict of interest statement

The authors declare that the research was conducted in the absence of any commercial or financial relationships that could be construed as a potential conflict of interest.

## Abbreviations

AIS: axon initial segment
ATP: Adenosine triphosphate
CMA: chaperone-mediated autophagy
EMC: ER membrane protein complex
EPCON: ER-peroxisome contact site
ER: endoplasmic reticulum
ERMES: ER mitochondria encounter structure
GPI: Glycophosphatidylinositol
IP3R, inositol 1, 4: 5-trisphosphate receptor
LD: lipid droplet
LPMC: lysosomal-peroxisome membrane contacts
MAM: mitochondria-associated membrane
MCU: mitochondrial low affinity calcium uniporter
MDVs: mitochondria-derived vesicles
MITO: mitochondria
NCLX: Na+/Ca2+ exchanger
ORPs: oxysterol-binding protein-related proteins
PAM: plasma membrane-associated membrane
PE: phosphatidylethanolamine
PS: phosphatidylserine
PIP: phosphatidylinositol phosphate
PI4P: phosphatidylinositol 4-phosphate
PKA: protein kinase
PM: plasma membrane
PML: promyelocytic leukemia
PMPs: peroxisomal membrane proteins
PO: peroxisome
PTS: peroxisomal targeting signal
ROS: reactive oxygen species
SERCA: ER sarcoplasmic/endoplasmic reticulum calcium ATPase
SOCE: store-operated calcium entry pathway
STIM: stromal-interacting molecule
UPR: unfolded protein response
VAPS: vamp-associated proteins.
